# Effect of Straw and Wood Ash on Soil Carbon Sequestration and Bacterial Community in a Calcareous Soil

**DOI:** 10.3389/fmicb.2022.926506

**Published:** 2022-07-18

**Authors:** Huili Zhao, Shakeel Ahmad, Nan Wu, Lizhu Suo, Xiaohong Tian, Ying Zhao, Jinyi Yu, Li Wang, Bingcheng Si

**Affiliations:** ^1^Key Laboratory of Coastal Eco Hydrological Process and Environmental Safety, School of Resources and Environmental Engineering, Ludong University, Yantai, China; ^2^Key Laboratory of Plant Nutrition and the Agri-Environment in Northwest China, College of Natural Resources and Environment, Ministry of Agriculture, Northwest A&F University, Xianyang, China; ^3^Guangxi Colleges and Universities Key Laboratory of Crop Cultivation and Tillage, College of Agriculture, Guangxi University, Nanning, China

**Keywords:** wood ash, bacterial community, carbon sequestration, straw incorporation, calcareous soil

## Abstract

Soil fertility can be improved by effectively utilizing agricultural waste. Straw can supply energy and wood ash adds nutrients to improve soil quality. However, few kinds of research have investigated the effect of wood ash and straw on soil carbon sequestration and the soil bacterial population, particularly in calcareous soils. The main goal of this current study was to quantify the impact of a combination of wood ash and straw on the indicators described above using stable δ^13^C isotope analyses by applying wheat straw to calcareous soil under a long-term C_4_ crop rotation. The incubation experiment included four treatments as follows: (i) no amendment (Control); (ii) amendment with wood ash (W); (iii) amendment with straw (S); and (iv) a combined amendment of straw and wood ash (SW). Our results showed that sequestration of soil inorganic carbon (SIC) in the SW and W treatments was higher (an average of 7.78%) than that in the S and Control treatments. The sequestered soil organic carbon (SOC) in the SW treatment was 1.25-fold greater than that in the S treatment, while there was no evident effect on the SOC content compared with straw alone. The microbial biomass carbon increased under SW by 143.33%, S by 102.23%, and W by 13.89% relative to control. The dissolved organic carbon increased under SW by 112.0%, S by 66.61%, and W by 37.33% relative to the control. The pH and electrical conductivity were higher in the SW and W treatments than in the S treatment and the control. The SW was conducive to maintaining soil enzymatic activities and bacterial diversity. Bacteroidetes and Actinobacteriota were dominant in SW, while the Acidobacteria phyla were dominant in the S treatment. The diversity of bacteria in the soil and community composition of the bacteria were predominantly assessed by the levels of water-soluble K, pH, and electrical conductivity. The incorporation of straw and wood ash is probably more effective at improving SIC and SOC sequestration and ameliorates the soil microhabitat.

## Introduction

It is very common to use agricultural wastes to boost soil fertility (Talukdar et al., 2018; Li et al., 2021). The principal soil organic carbon (SOC) source in farming systems is organic manure and crop residues (Liang et al., 2021; Yang et al., 2022). In some geographical regions, organic manure is commonly for cash crops, and straw return effectively improves SOC sequestration in farmland systems. Large amounts of wood ash produced from wood industries are one of the challenging processes that the companies face (Juárez et al., 2013). Recycling biomass ash in the agricultural industry can provide use for biomass ash by recycling it, which could settle the issues of disposal and reduce the need for commercial fertilizer to use on crops.

In one previous study, Zhao et al. (2017, 2018) reported that combining wood ash and straw could minimize CO_2_ emissions, which are largely due to inorganic carbon formation. Nonetheless, the impact of combining crop straw and wood ash on SOC turnover in calcareous soil has not been fully understood yet. Previous studies have found that using mineral amendments, such as wood ash, to apply to the soil resulted in carbon with a high specific surface area and high contents of metal oxides that can help organic residues to raise the amount of C in the soil (Reed et al., 2017; Muhammad et al., 2021a). Lei et al. (2019) have reported that both fly ash composite and organic fertilizer can be used to improve reclaimed soil. It is not clear whether wood ash promotes or inhibits C mineralization in native SOC, particularly when combined with straw, and also, little is known regarding the combination of straw-derived C sequestered in calcareous soil.

In addition, it is highly important to investigate the modifications induced by the addition of straw and wood ash to the soil microbial community. Bacterial diversity and abundance are the foundations for soil productivity in agricultural systems (Fierer and Jackson, 2006; Fierer et al., 2007; Bhat, 2013; Muhammad et al., 2021b). So far, most of the studies have focused on the addition of wood ash to acidic agricultural and forest soils. In addition, some of the studies that analyzed soil biological characteristics produced conflicting reports on the usage of wood ash in the components of soil biology. Peltoniemi et al. (2016) showed that mycorrhizae and wood-decomposing fungi appeared owing to the higher pH and additional nutrients caused by wood ash fertilization in acidic soil. Noyce et al. (2016) observed that wood ash addition has only minimal effects on the composition of the soil microbial community in sites across two distinct global forest biomes. Straw provides energy for the soil, and wood ash provides nutrients for the soil. However, few studies have explored the combination of microbial communities, especially in calcareous soils.

An incubation experiment was conducted on the chemical properties, soil carbon sequestration, and bacterial community in calcareous agricultural soil after amendments with straw and wood ash. Our goals were to (i) investigate the soil carbon sequestration variations after adding a combination of wood ash and straw, (ii) measure the enzyme activities and soil bacterial communities after the adjunction of a combination of straw and wood ash, and (iii) illustrate the interactions that take place between soil bacterial communities and carbon sequestration. We hypothesized that the adjunction of straw and wood ash would enhance the nutrient supply and alter the soil pH, consequently increasing the activities of soil enzymes and the relative diversity and abundance of the soil bacteria, resulting in greater amounts of soil carbon sequestration.

## Materials and Methods

### Characterization of Wood Ash, Wheat Straw, and the Soil

We randomly collected soil samples from several sampling points of the top 20-cm layer (One composite soil sample) in the Changwu Agricultural and Ecological Experimental Station (35.14° N, 107.40° E; 1,152 m a.s.l.) on the Loess Plateau in northwestern China. This field has been cultivated under a monocropped planting of maize, a C_4_ crop, for at least 12 years. When the soil was air-dried and well mixed, dislodged crop residues were passed through a sieve of 2 mm. The soil with a δ^13^C value of −19.5% was classified as a Cumuli-UsticIsohumosol by the USDA classification system and had a silty loam texture. The soil samples contained 8.9 g kg^–1^ SOC, 1.2 g kg^–1^ total nitrogen (TN), 163.0 mg kg^–1^ microbial biomass carbon (MBC), 32.4 mg kg^–1^ dissolved organic carbon (DOC) as determined in the methods below, 18.4 mg kg^–1^ of available phosphorus, and 152.3 mg kg^–1^ of available potassium, pH (H_2_O) 7.9 (Gong et al., 2007).

Wheat straw (a C_3_ plant) harvested from the Doukou Experimental Station of Northwest A&F University (Shaanxi, China) was dried in a 60°C oven and cut into small pieces that were approximately 2 cm long before they were mixed with the soil. The straw had total C and N contents of 456.1 and 6.8 g kg^–1^ (66 C:N). The wheat straw had a δ^13^C value of −27.5%.

The wood ash was the ash of kiwifruit branches after burning. The ash was then dried for 24 h in a 60°C oven and mixed before use and analysis. The δ^13^C value of the wood ash was −26.4‰ (SE). The total carbon from the wood ash (as described in the method below) and the organic C were 39.3 g kg^–1^ and 3.2 g kg^–1^, respectively, and the soil inorganic carbon (SIC) added from the wood ash was 432 mg C kg^–1^ soil. This study comparatively assessed the composition of the chemicals in the wood ash used in these experiments concerning the wood ash used in other studies to evaluate to what extent the results of this study could be extrapolated to other wood ash and soils (Table 1).

**TABLE 1:** Elemental composition of the wood ash used in this study and those from other studies.

Parameters	Wood ash^δ^	Delgado-Baquerizo et al., 2013	Klemedtsson et al., 2010	Reed et al. (2017)
pH	12	13	-	11
EC^※^	25	4	-	17
Ca^#^	310	250	209	194
K^#^	125	26	35	79
Si^#^	58	57	43	-
Mg^#^	59	27	24	-
P^#^	49	7	13	17
Al^#^	24	-	19	-
S^#^	16	87	6	6
Fe^#^	17	10	10	-
Na^#^	8	-	5	-
Cl^#^	7	-	-	3
Ti^#^	2	-	1	-
Sr^#^	1	-	-	-
Cr^&^	400	38	-	-
Zn^&^	400	410	-	1,369
Cu^&^	200	120	-	197
Rb^&^	200	-	-	-

*^δ^The wood ash that was used in this study.*

*^※^The unit was dS m^–1^.*

*^#^The unit was g kg^–1^.*

*^&^The unit was mg kg^–1^.*

### Experimental Design

The incubation experiment was conducted at 25°C in a dark laboratory, which included four treatments with three replicates: (1) no amendment (Control), (2) amendment with wood ash (W, 12 g kg^–1^ soil), (3) amendment with wheat straw (S, 10 g kg^–1^ soil), and (4) amendment with combined wheat straw and wood ash (SW, 10 g straw kg^–1^ and 12 g wood ash kg^–1^ soil). The soils (250 g dry weight equivalent) were mixed with straw and/or wood and then incubated in 12 plastic jars 15 cm high and 9 cm in diameter. Diammonium phosphate and urea were suspended in deionized water before addition as a solution (159 mg N kg^–1^ dry soil, which corresponded to 357 kg^–1^ ha^–1^ in field conditions, and 185 mg P_2_O_5_ kg^–1^ dry soil, which corresponded to 416 kg^–1^ ha^–1^ in field conditions). The soil moisture (or soil-straw and/or wood ash mixture) in the jars was adjusted to 70% of field water holding capacity by weighing and adding distilled water and maintained throughout the experiment.

### Measurement of Soil CO_2_ Effluxes and Soil Characteristics

The entire process for the incubation and determination of CO_2_ and its calculations was conducted as described by Zhao et al. (2017) and Zhao et al. (2018). Soil CO_2_ emissions were determined and calculated on days 2, 3, 4, 5, 7, 10, 15, 20, 25, 35, 45, 65, 95, and 118 after incubation, and the small plastic bottle containing NaOH was exchanged for a new one. The trapped CO_2_ was precipitated with 1.0 mol L^–1^ BaCl_2_, and then titrated with standard 0.1 mol L^–1^ HCl to quantify the released CO_2_. All the samples pass through a 2 mm sieve, mixed evenly, and separated into three subsamples at the end of incubation. One portion was stored at 4°C for analysis; one was dried in the open air for soil analysis. The other was stored at −80°C for DNA extraction and subsequent molecular analyses. The SOC was determined using the wet-oxidation-redox titration method (Lu, 2000). We measured the δ^13^C natural abundance of the wood ash, SOC, and wheat straw as described by Zhao et al. (2017) and Zhao et al. (2018). After the incubation with straw, any partially decomposed plant residue was removed from the soils using a dry-sieving/winnowing procedure. One gram of soil was preconditioned for 12 hours with 10 mlL of 1 M HCl to extricate the carbonate. We quantified the amount of straw-derived C (newly formed SOC) using a two two-end-member mixing model (Balesdent et al., 1987).


(1) $${\text{f}}_{\text{new}}\left(\%\right)=\left(\delta^{13}C_{\mathrm{SOC}-a}-\delta^{13}C_{\mathrm{SOC}-b}\right)/\left(\delta^{13}C_{\text{material}}-\delta^{13}C_{\mathrm{SOC}-b}\right)$$

where f_new_ (%) is the ratio of SOC from wheat straw to total SOC; δ^13^C_soc–a_ is the δ^13^C of SOC in the amended soil samples following incubation; δ^13^C_soc–b_ is the δ^13^C of SOC in non-amended soil samples before incubation, and the δ^13^C_material_ is δ^13^C of straw an d straw plus the wood ash mixture.


(2) $$\text{Newly}\text{SOC formed}=f_{\mathrm{new}}\left(\%\right)\times \mathrm{Total}\,\text{SOC}$$

The amount of sequestered SOC was calculated as follows (Kirkby et al., 2014):


(3) $$\mathrm{Sequestered}\,\mathrm{SOC}\,\left(\mathrm{mg}\,{\text{kg}}^{-1}\right)={\mathrm{SOC}}_{a}-{\mathrm{SOC}}_{b}$$

SOC_*a*_ and SOC_*b*_ are the amounts of SOC with straw amendments added after the incubation and those without straw before the incubation, respectively.


(4) Native SOC mineralization=Newly SOC⁢formed-Sequestered⁢SOC

The soil MBC was measured using a chloroform-fumigation as an extractant (Vance et al., 1987). The DOC was extracted from 10 g of moist soil at 25.8°C using a ratio of 1:2.5 soils to water (Jiang et al., 2006). The soil TN was determined as Huang et al. (2007) described. The soil pH and electrical conductivity (EC) were determined in a 1:5 slurry of soil:water (w/w). Mineral nitrogen (Min-N) included ammonium nitrogen (AN) and nitrate nitrogen (NN). The soil KCl-extractable AN and NN were extracted with 2 M of KCl, steam distillation, and titration for analysis. The SIC was determined as described by Bao (2008). The total carbon of wood ash and soil organic C were measured using an elemental analyzer (Vario MAX; Elementar, Germany). The activity of catalase was determined by titrating with KMnO_4_ using H_2_O_2_ as the substrate (Guan, 1986). Invertase activity was assayed by titration with sodium thiosulfate as described by Guan (1986). Dehydrogenase (EC 1.1) activity was estimated by reducing 2,3,5-triphenylterazolium chloride (Casida et al., 1964). The activities of three hydrolytic enzymes, β-glucosidase, β-1,4-xylosidase, and cellobiohydrolase, were measured with some buffer modifications using a fluorescent microplate enzyme assay (DeForest, 2009).

### Soil Total Community DNA Extraction and 16S rRNA Sequencin*g*

Samples of the total genomic DNA were extracted using an OMEGA Soil DNA Kit (D5625-01) (Omega Bio-Tek, Norcross, GA, United States) following the manufacturer’s instructions and then stored at −20°C for additional analysis. The quantity and quality of the extracted DNAs were measured using a NanoDrop ND-1000 spectrophotometer (Thermo Fisher Scientific, Waltham, MA, United States) and agarose gel electrophoresis, respectively. High-throughput sequencing of the bacterial 16S rRNA genes from the soil samples was performed by the Xuan Chen Biological Technology Co., Ltd. (Shaanxi, China). The V3–V4 regions of the bacterial 16S rRNA genes were amplified using the forward primer 338F (5′-ACTCCTACGGGAGGCAGCA-3′) and the reverse primer 806R (5′-GGACTACHVGGGTWTCTAAT-3′).

### Statistical Analysis

ASV-level alpha diversity indices, such as the Chao1 richness estimator, Shannon diversity index, Observed reads, and Good’s coverage, were calculated using the ASV table in QIIME2 and visualized as box plots. Graphs were prepared using SigmaPlot 12.5 (SYSTAT, San Jose, CA, United States). A correlation analysis was conducted using HemI. Distance-based redundancy analysis with CANOCO 5.0 was used to analyze the correlations between the physicochemical and biological properties of the soil and bacterial community structure. Variance partition analysis (VPA) was used to quantify the contribution rates of the major bacterial communities to SOC using the “Vegan” program in R 3.5.1 statistical software. A one-way ANOVA was used to assess the effects of wood ash and straw on soil chemical characteristics, soil carbon sequestration, and soil bacterial diversity and community. All ANOVAs were conducted using a general linear model (GLM), and significant differences between means were identified using Fisher’s protected least significant difference (LSD) test at *p* ≤ 0.05.

All of the sequence data were deposited in the NCBI Sequence Read Archive (SRA) database under accession number PRJNA827340.

## Results

### Soil C Mineralization, Soil Chemical Characteristics and Enzyme Activities

**FIGURE 1:**
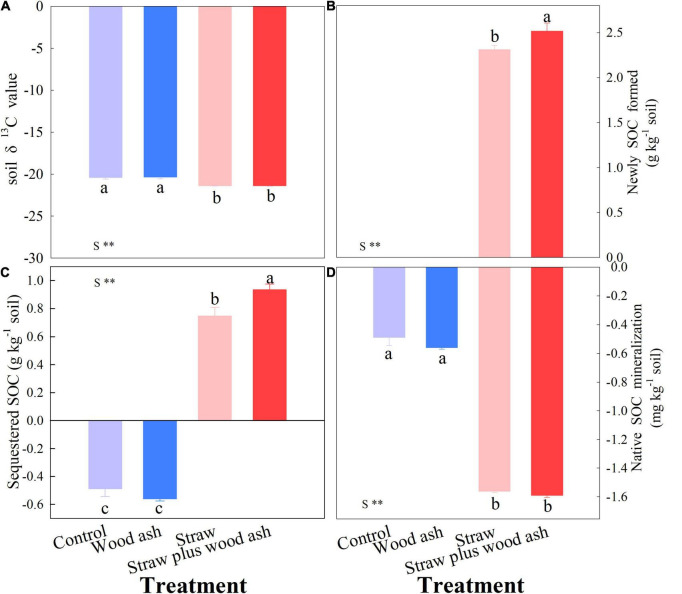
Effects of wood ash and straw treatments (addition or no addition) on the value of soil δ^13^C **(A)**, on the net change in soil organic carbon (SOC) sequestration **(B)**, on newly SOC formed **(C)** and native SOC mineralization **(D)** at the end of incubation. Data (means ± SE, *n* = 3) followed by different letters indicate significant differences among the treatments at *p* < 0.05. See formulas 2, 3, and 4 for the calculation of native SOC minimization. **Indicated that there was significant difference between straw and other treatments.

The cumulative CO_2_-C emissions (namely all of the CO_2_ measurements) decreased by 11.1% in the treatment with wood ash compared with that of soil alone. Moreover, the cumulative CO_2_-C emissions decreased by 6.0% in straw plus wood ash treatment relative to the addition of straw alone (Table 2). The SOC between straw alone and straw plus wood ash treatments did not differ significantly. However, the wheat straw addition decreased soil δ^13^C value (Figure 1A; the contents of newly and sequestered SOC increased by 8.8 and 54.7% in the straw plus wood ash relative to straw treatment, respectively (Figures 1B,C). Straw addition promoted native SOC mineralized (Figure 1D). On average, SIC content (removed the SIC in wood ash) showed an increase of 218 mg C kg^–1^ soil under the wood ash treatments with or without straw relative to those under the control and straw treatment (Table 2). This study showed that wood ash amendment significantly increased SICS content. The DOC and MBC levels for the straw plus wood ash, straw, and wood ash treatments increased by 112, 66, and 37%; and 143, 102, and 14%, respectively, with significant differences compared with the control treatment (Table 2). The high soil pH following wood ash amendment increased the solubility of organic matter compared to that of organic matter in soil samples with no amendment, which resulted in higher DOC content in the wood ash-amended soil.

**TABLE 2:** Effects of wood ash, maize straw residue, and their interaction on soil chemical characteristics and enzyme activity after 118 days of incubation.

	Control	Wood ash	Straw	Straw plus wood ash	S	W	S × W
Cumulative CO_2_-C (g kg^–1^)	0.9 ± 0.01c	0.7 ± 0.01d	5.0 ± 0.01a	4.7 ± 0.01b	< 0.001	< 0.001	< 0.001
SOC (g kg^–1^)	8.42 ± 0.10	8.35 ± 0.20	9.66 ± 0.10	9.84 ± 0.20	< 0.001	0.442	0.108
SIC (g kg^–1^)	8.35 ± 0.30	9.00 ± 0.20	8.35 ± 0.10	9.00 ± 0.20	0.952	< 0.001	0.931
MBC (mg kg^–1^)	180 ± 1.5	205 ± 1.4	364 ± 4.2	438 ± 1.9	< 0.001	< 0.001	< 0.001
DOC (mg kg^–1^)	50.9 ± 2.8d	69.9 ± 3.0c	84.8 ± 5.0b	107.9 ± 3.7a	< 0.001	< 0.001	0.362
Soil moisture	0.246 ± 0.0b	0.247 ± 0.0b	0.259 ± 0.0a	0.258 ± 0.0a	< 0.001	0.922	0.514
Water soluble Ca (mg kg^–1^)	286 ± 5.1ab	318 ± 31.9a	282 ± 40.2ab	249 ± 16.5b	0.047	0.963	0.070
Water soluble K (mg kg^–1^)	0.3 ± 0.1b	20.3 ± 0.6a	23.6 ± 2.4a	22.5 ± 4.1a	< 0.001	< 0.001	< 0.001
Water soluble Mg (mg kg^–1^)	21.6 ± 8.2b	28.9 ± 6.7ab	27.5 ± 17.0ab	44.5 ± 13.0a	0.159	0.116	0.495
pH	7.94 ± 0.07	8.07 ± 0.02	8.04 ± 0.10	8.16 ± 0.02	0.054	0.042	0.634
EC (us/cm)	180 ± 1.5c	619 ± 34.9a	379 ± 12.8b	608 ± 33.5a	< 0.001	< 0.001	< 0.001
TN (g kg^–1^)	1.2 ± 0.0b	1.2 ± 0.0b	1.3 ± 0.0a	1.3 ± 0.0a	< 0.001	0.130	0.614
Min-N (mg kg^–1^)	79.8 ± 0.5a	80.1 ± 0.1a	61.9 ± 1.3b	58.6 ± 3.3c	< 0.001	0.037	0.030
Enzyme activity							
β-1,4-xylosidase (nmol h^–1^ g^–1^)	7.0 ± 0.1b	5.8 ± 0.6b	10.8 ± 2.0a	9.7 ± 1.6a	< 0.001	0.144	0.953
β-glucosidase (nmol h^–1^ g^–1^)	18.3 ± 0.5b	16.1 ± 1.0b	23.6 ± 2.1a	22.4 ± 0.3a	< 0.001	0.040	0.466
Cellobiohydrolase (nmol h^–1^ g^–1^)	6.7 ± 0.4b	4.6 ± 0.4c	8.8 ± 0.2a	7.4 ± 0.5b	< 0.001	< 0.001	0.120
Invertase (mg g^–1^ d^–1^)	52.1 ± 5.2b	53.5 ± 10.6b	59.7 ± 3.4ab	69.1 ± 1.2a	0.012	0.172	0.298
Catalase (mg g^–1^ d^–1^)	1.2 ± 0.0a	0.9 ± 0.1b	1.1 ± 0.0a	1.2 ± 0.1a	0.008	0.017	< 0.001
Dehydrogenase (mg kg^–1^ h^–1^)	7.2 ± 0.8c	5.6 ± 0.6d	16.8 ± 1.3b	20.7 ± 0.6a	< 0.001	0.042	< 0.001

*Data are the means ± SE, n = 3. MBC, microbial biomass carbon; DOC, dissolved organic carbon; SIC, soil inorganic carbon; TN, total nitrogen; EC, electrical conductivity; Min-N, mineral nitrogen. S, straw; W, wood ash. Different lowercase letters indicate significant differences among the treatments at p < 0.05.*

The highest soluble K was found in the soil amended with straw, followed by treatment with wood ash, indicating that the availability of potassium from the wood ash was relatively high and was even equivalent to the soil in which mineral K sulfate fertilizer was used applied (Table 2). The data showed that the soil pH differed significantly among the treatments. The application of wood ash brought about an average augment of pH from 7.9 in the control and straw treatments to 8.2 in wood ash and straw plus wood ash treatments (Table 2). The EC in wood ash, and straw plus wood ash treatments was 33.7 and 30.4% higher than that of the control, respectively (Table 2).

The effects of the addition of straw and the interaction of application of straw × wood ash on catalase and dehydrogenase activity were significant (Table 2). Only adding wood ash decreased the activities of cellobiohydrolase, catalase, and dehydrogenase. However, integrating straw with wood ash maintained them at the original level. Surprisingly, adding wood ash alone increased invertase activity, and integrating straw with wood ash further enhanced their activities.

The correlation analysis revealed that the DOC, S_moisture_, K, Mg, and TN were significantly and positively correlated with each other and soil enzyme activities, while significantly negatively correlated to M_in_-N (Table 3). Similarly, the invertase, catalase, dehydrogenase, β-1,4-xylosidase, cellobiohydrolase, and β-glucosidase activities were also highly positive and significantly correlated with each other and negatively correlated to M_in_-N (Table 3).

**TABLE 3:** Pearson’s correlation analysis for soil enzymes and physio-chemical properties.

	Min-C	DOC	S_moisture_	Ca	K	Mg	EC	TN	Min-N	Invertase	Catalase	Dehydrogenase	BXYL	BG
DOC	0.86**													
S_moisture_	0.95**	0.82**												
Ca	–0.40	–0.37	–0.29											
K	0.63*	0.75**	0.67*	0										
Mg	0.57*	0.90**	0.57*	–0.38	0.61*									
EC	0.19	0.58*	0.27	0.03	0.76**	0.74**								
TN	0.86**	0.86**	0.89**	–0.35	0.73**	0.70**	0.44							
Min-N	−0.95**	−0.95**	−0.88**	0.46	−0.63*	−0.75**	–0.32	−0.84**						
Invertase	0.66*	0.79**	0.68*	–0.08	0.47	0.73**	0.43	0.63*	−0.75**					
Catalase	0.49	0.34	0.42	–0.4	–0.20	0.21	–0.45	0.25	–0.51	0.43				
Dehydrogenase	0.95**	0.93**	0.91**	–0.41	0.57*	0.74**	0.27	0.88**	−0.97**	0.77**	0.58*			
BXYL	0.86**	0.67*	0.84**	–0.22	0.49	0.34	–0.05	0.65*	−0.80**	0.53	0.63*	0.82**		
CBH	0.93**	0.71**	0.89**	–0.35	0.45	0.41	–0.03	0.73**	−0.85**	0.52	0.63*	0.88**	0.91**	
BG	0.83**	0.49	0.73**	–0.37	0.17	0.15	–0.33	0.50	−0.72**	0.37	0.67*	0.73**	0.81**	0.90**

*DOC, dissolved organic carbon; S_moisture,_ soil moisture; Min-N, mineral nitrogen; TN, total nitrogen; EC, electrical conductivity; BXYL, β-1,4-xylosidase; CBH, Cellobiohydrolase; BG, β-glucosidase; Min-C, C mineralization.*

*Indicates P < 0.05, ** indicates P < 0.01.

### Species Richness and Diversity of Bacteria

**FIGURE 2:**
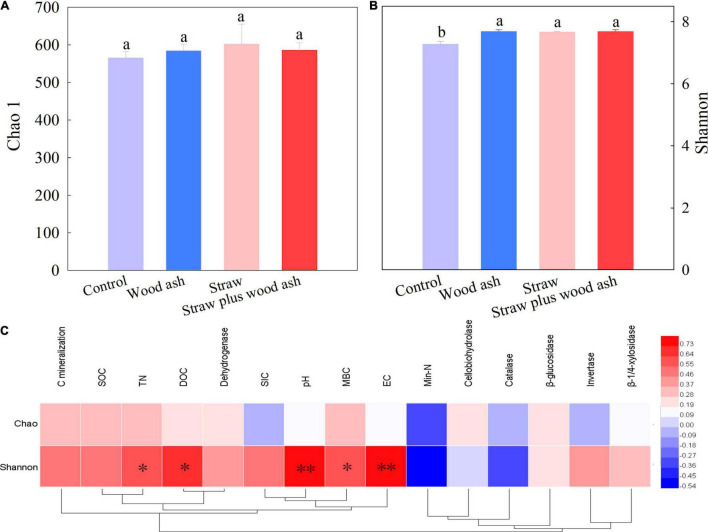
**(A,B)** The richness of soil bacterial community and the diversity of soil for each treatment after incubation for 118 days. **(C)** Pearson’s correlation coefficients between alpha diversity and soil properties. *P < 0.05; **P < 0.01. Data (means ± SE, *n* = 3) followed by different letters indicate significant differences among the treatments at *p* < 0.05.

The Chao1 indices did not identify any significant differences among the treatments. The Shannon index of the wood ash, straw, and straw plus wood ash treatments was higher than the Control (Figures 2A,B). In contrast, the Shannon index significantly positively correlated with the DOC (*r* = 0.67, *P* < 0.05), MBC (*r* = 0.59, *P* < 0.05), TN (*r* = 0.60, *P* < 0.05), pH (*r* = 0.80, *P* < .01), and EC (*r* = 0.82, *P* < 0.01) (Figure 2C), suggesting that the diversity of soil bacteria was affected by soil labile carbon and pH among the addition of exogenous substances.

### Bacterial Community Structure

**FIGURE 3:**
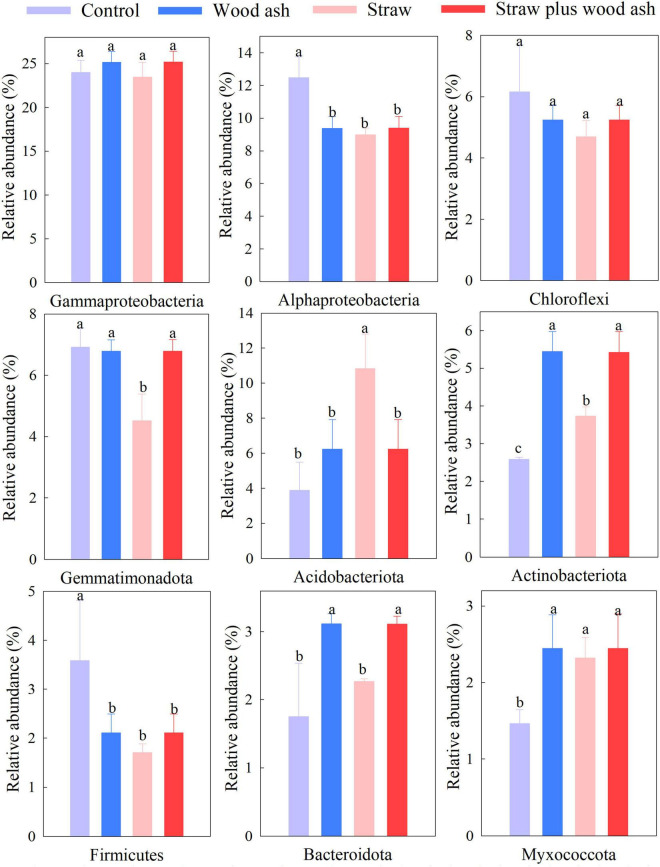
The relative abundance of levels of different phyla in this study. Control, soil alone; W, addition of wood ash; S, addition of wheat straw; SW, addition of wheat straw plus wood ash. Data (means ± SE, *n* = 3) followed by different letters indicate significant differences among the treatments at *p* < 0.05.

The Illumina platform analysis was adopted to clarify the soil’s bacterial community structure and composition. *Proteobacteria*, *Chloroflexi*, and *Gemmatimonadetes* were the three dominant phyla, and the abundances of phyla of *Acidobacteriota*, *Actinobacteriota*, and *Bacteroidetes* differed significantly between the wood ash, straw plus wood ash, and straw alone treatments, thus indicating that the change of soil pH changed the relative abundance of bacteria (Figure 3).

Twelve samples were investigated at the 90% similarity level (three in each of the four treatments) and divided into two main groups (Supplementary Figure 1). The straw, straw plus wood ash treatments grouped with the control, and only the wood ash amendment formed a group. Moreover, the three replicate samples of each treatment were most closely related to each other (Supplementary Figure 1). Therefore, these data suggest an important role for straw in alleviating bacterial community changes caused by amendment with wood ash. A Venn diagram demonstrated that the OTUs differed among four treatments (Supplementary Figure 2). OTUs ranged from 156 (straw plus wood ash) to 336 (straw). Only 1,468 out of a total of 3,841 OTUs were shared by all four treatments (Supplementary Figure 2).

**FIGURE 4:**
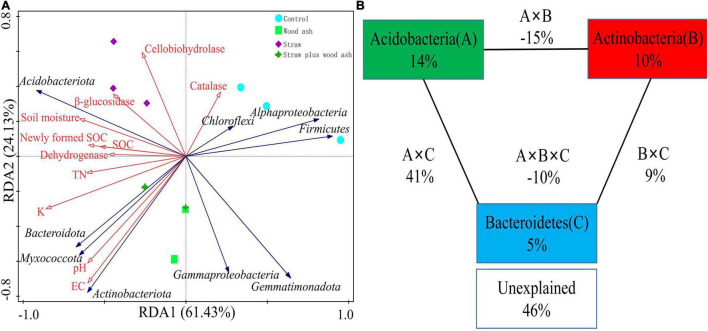
Distance-based redundancy analysis to show the correlations between the bacterial phyla and chemical characteristics **(A)** and variance partition analysis **(B)** between the sequestered SOC of bacterial phyla from different treatments.

**FIGURE 5:**
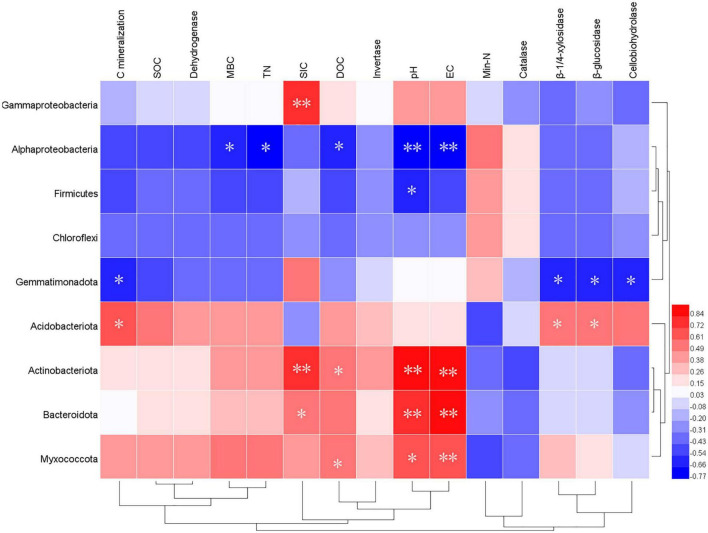
Pearson’s correlation between soil characteristics and the relative abundance of bacterial groups (phylum level). **Correlation is significant at the 0.01 level. *Correlation is significant at the 0.05 level.

Distance-based redundancy analysis (db-RDA; Figure 4A) and Pearson’s correlation coefficient (Figure 5) were performed to examine the relationship between chemical characteristics, enzyme activities, and bacterial community composition. The bacterial communities shifted with the addition of straw and wood ash along RDA1 and RDA2, respectively. The study also showed that the six soil samples treated with wood ash and straw plus wood ash were closely related and differed significantly from the samples treated with straw alone and those from the Control (Figure 4A). Environmental factors significantly correlated with the composition of the bacterial community. These factors explained 85.5% of the bacterial community variation (Figure 4A). Among all the environmental variables examined, the water-soluble K (*F* = 9.3, *P = 0.*002), soil EC (*F* = 5.4, *P = 0*.008), pH (*F* = 4.6, *P = 0.*01), DOC (*F* = 3.2, *P = 0*.05) and TN (*F* = 3.1, *P = 0*.04) explained 48.1, 35.3, 31.4 24.0, 24.0 and 23.5% of the variation of communities, respectively, indicating that these variables have an important role in altering the bacterial communities (Figure 4A). A VPA analysis indicated that the relative abundances of Acidobacteria, Actinobacteria, and Bacteroidetes contributed 14, 10, and 5% to the variation in the content of SOC, respectively (Figure 4B). The total rate of contribution of each variable and its interaction with the variation in the content of SOC was 54%.

**FIGURE 6:**
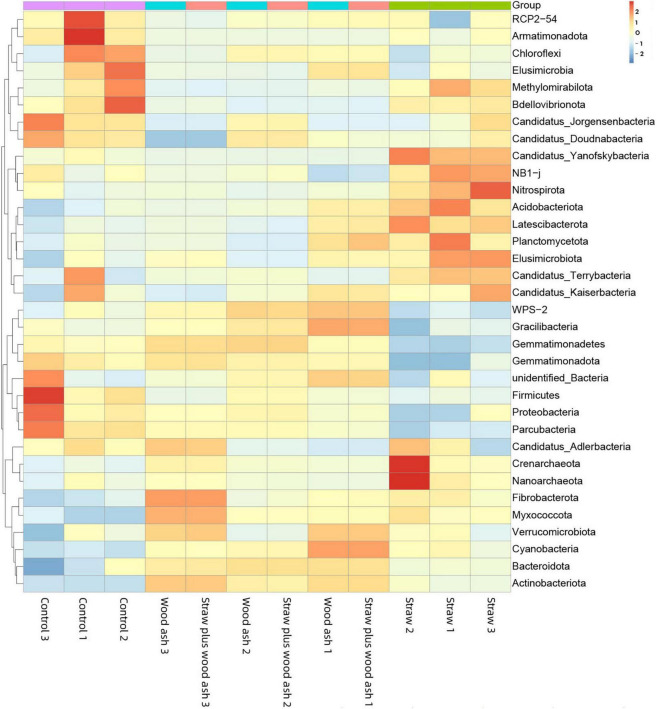
Microbial community composition of top 50 abundant operational taxonomic units (OTUs) and their abundance level with cluster analysis. The dendrogram linkages and OTUs distances are not phylogenetic but based upon the reads number. The legend and scale represent the abundance of OTUs (Y-axis) within each sample (X-axis). The red indicates more abundant phyla and the blue represents less abundant phyla in the corresponding samples.

Furthermore, the relationship between the relative abundance (> 1%) of the soil characteristics and phyla is shown in Figure 5. The sub-phylum *Alphaproteobacteria* negatively correlated with the contents of DOC, MBC content, pH, and EC. The *Gemmatimonadetes* had a significantly negative relationship with the cumulative CO_2_-C (*P* < 0.05), β-1, 4-xylosidase (*r* = 0.81, *P* < 0.01), β-glucosidase (*r* = 0.84, *P* < 0.01), and cellobiohydrolase (*r* = 0.68, *P* < 0.05). In addition, the *Acidobacteriota* had a clear opposite trend of a positive correlation. Furthermore, *Actinobacteriota, Bacteroidetes*, and *Myxococcota* positively correlated with the soil pH and EC to a remarkable extent (Figure 5). The data on community composition for each bacterial phyla were clustered based on abundance distribution or degree of similarity. The bacterial phyla, straw, and wood ash were graded independently and presented using heat maps based on the clustering results. Color gradients were adopted to differentiate between phyla with high and low abundance (Figure 6).

## Discussion

### Effects of the Application of Wood Ash and Straw on the Mineralization of Soil C, Soil Chemical Properties, and Enzyme Activities

In some studies, the effects of wood ash on the CO_2_ emissions of forest peat land increased owing to an increase in the pH and nutrient contents of the soil (Moilanen et al., 2012). In contrast, Klemedtsson et al. (2010) found that amendment with wood ash could be an appropriate mitigation measure for CO_2_ emissions from a spruce forest. This study found that the application of wood ash significantly decreased soil CO_2_ emission in the order of straw > straw plus wood ash > control > wood ash (Table 2). The decrease in CO_2_ emissions in straw plus wood ash treatment was much higher than the SIC content, which could be partly owing to the formation of SIC and partly owing to the conversion of more straw to newly formed SOC (e.g., the MBC and DOC). Those results indicated that the addition of wood ash did reduce CO_2_ emissions in acidic and alkaline soils. In addition, the data showed that the addition of wood ash promoted the formation of new organic carbon and the retention of net organic carbon but did not affect the mineralization of native SOC. This result suggests that when wood ash was present, the increase in the content of SOC might be owing to the progressive breakdown and transformation of straw by the enhanced dehydrogenase and invertase activities. However, this result was consistent with that of Reed et al. (2017), who noted that wood ash brought about a lasting beneficial impact on SOM turnover owing to the CEC and the ability of the specific surface area of wood ash to chemically stabilize the SOM.

The addition of straw and wood ash enhanced the contents of MBC and DOC relative to treatment with the addition of straw (Table 2), which may be directly affected by the enhanced enzyme activity and a change in the pH of the soil. The applications of wood ash increased the pH, which was influenced largely by the dissolution of several oxides, hydroxides, carbonates, and bicarbonates contained in wood ash (Vassilev et al., 2013). Consistent with the change in pH, the increase in soil EC after wood ash was added was owing to the dissolution of wood ash during cultivation that contained high amounts of alkaline ions (Table 2). The addition of straw and wood ash affected mineral nitrogen with the order: straw plus wood ash < straw < wood ash ≈ Control (Table 2). This indicated the combination increased the immobilization of mineral nitrogen, which may further promote soil organic nitrogen formation, improve the inorganic nitrogen sequestration, and reduce the leaching loss of nitrogen (Muhammad et al., 2022b).

The fact that catalase and dehydrogenase were only reduced by wood ash could be attributed to at least two reasons. First, the wood ash adsorbed the substrate used by catalase and dehydrogenase and inhibited the progress of enzyme reaction. Second, the high pH of the wood ash itself could also change the microbial community, resulting in a decrease in the secretion of catalase and dehydrogenase. The promotion of enzyme activity by straw plus wood ash was owing to the high pH caused by added wood ash to promote the hydrolysis of straw to produce more catalase and dehydrogenase reaction matrix (Gömöryová et al., 2016; Bang-Andreasen et al., 2017). The invertase activity was enhanced underwood ash compared with the control. This was probably caused by the increase in pH due to wood ash, which aided in releasing more sucrose from the straw or soil (Table 2).

### Effects of the Application of Wood Ash and Straw on Soil Bacterial Diversity

There were no differences in bacterial species richness among the treatments. Toke et al. (2017) recently found that the EC and pH had an adverse impact on bacterial diversity, while our results are contrary to theirs; we found that the MBC, DOC, pH, and EC have an important role in the increase in bacterial diversity based on Pearson’s correlation coefficients (Figure 2). Indeed, the decomposition of C provides energy for most soil microorganisms, and recent studies found that soil bacterial diversity is driven by soil C storage (Delgado-Baquerizo et al., 2013; Maestre et al., 2013; Muhammad et al., 2022a). However, the long-term experiment combining straw with wood ash used in the field merits further study.

### Effects of the Application of Wood Ash and Straw on the Soil Bacterial Community Structure

Soil pH is a primary factor regulating microbial community structure. High pH values were favorable to *Bacteroidetes* and *Actinobacteriota* (Lauber et al., 2009; Rousk et al., 2010). *Bacteroidota* and *Actinobacteriota* increased relative abundance following the straw plus wood ash and wood ash alone relative to the straw alone (Figure 3). Noyce et al. (2016) indicated that applying wood ash could increase the abundance of *Bacteroidota*. The possible increase in DOC and the more neutral pH at applications of 22 t ha^–1^ of ash improves the conditions for the growth of the copiotrophic *Bacteroidota*. *Acidobacteriota* was enhanced by the straw alone and is a heterotroph that can utilize extensive carbon sources (Ward et al., 2009; Langille et al., 2013), thus, displaying a crucial role in the carbon cycle. The control treatment, in concert with the lack of effects of the application of degradable C, can help explain the lower bacterial numbers and the increased abundance of *Firmicutes* in this treatment that lacked amendments. The dominant phylum *Proteobacteria* maintains a high relative abundance (32.4–36.5%) throughout all the treatments (Figure 3), which the general resistance of Proteobacteria might explain to environmental changes (Barnard et al., 2013). This study found that the highest percentage of *Alphaproteobacteria* was identified in the treatment that lacked amendments (lower pH), which is inconsistent with those of Ding et al. (2016), who found that the populations of *Alphaproteobacteria* negatively correlated with the soil pH. In addition, soil moisture is a critical factor in governing soil community composition.

## Conclusion

This study showed that the addition of wood ash to agricultural soil could obviously vary in its chemical and microbial characteristics. Treatments with wood ash (straw plus wood ash and wood ash) increased the content of SIC relative to other treatments. The amounts of newly SOC formed and sequestered SOC in the straw plus wood ash treatment were greater than those in the straw treatment. Our results confirmed that adding exogenous substances enhanced the bacterial diversity compared with that of the control. Furthermore, a redundancy analysis showed that the EC and water-soluble K are the most influential factors that determine the structure of soil bacterial communities. In conclusion, integrating straw with wood ash had the same effect on bacterial diversity relative to the addition of straw alone. However, the former might be conducive to SOC and SIC sequestration.

## Data Availability Statement

The data presented in this study are deposited in the https://www.ncbi.nlm.nih.gov/search/all/?term=PRJNA827340 repository, accession number BioProject: PRJNA827340.

## Author Contributions

HZ, SA, NW, XT, BS, and YZ designed all the experiments and wrote the manuscript. HZ, XT, JY, and LW were responsible for performing the field and lab experiments. HZ, LS, and SA wrote, reviewed, and edited the manuscript. All authors analyzed all data and discussed the results.

## Conflict of Interest

The authors declare that the research was conducted in the absence of any commercial or financial relationships that could be construed as a potential conflict of interest.

## Publisher’s Note

All claims expressed in this article are solely those of the authors and do not necessarily represent those of their affiliated organizations, or those of the publisher, the editors and the reviewers. Any product that may be evaluated in this article, or claim that may be made by its manufacturer, is not guaranteed or endorsed by the publisher.
