# Supplementary material for: Effect of Straw and Wood Ash on Soil Carbon Sequestration and Bacterial Community in a Calcareous Soil
Source: Front Microbiol. 2022 Jul 18;13:926506. doi: 10.3389/fmicb.2022.926506 (PMC9339994; doi:10.3389/fmicb.2022.926506)
Supplement: Supplementary file 1 [file Data_Sheet_1.docx]

**TABLE S1** Reads, Observed OTUs, the bacterial community richness and diversity of soil for each treatment after the 118 d incubation.

| Treatment | Reads | OTUs^b^ | Coverage |
| --- | --- | --- | --- |
| Control | 34974±2107 a | 558±8 a | 0.9960±0.001 a |
| Wood ash | 37917±3038 a | 592±24 a | 0.9967±0.001 a |
| Straw | 34805±481 a | 590±37 a | 0.9970±0.001 a |
| Straw plus wood ash | 37927±3051 a | 592±24 a | 0.9967±0.001 a |
| *P* (LSD_0.05_) values in ANOVAs test | | | |
| Straw (S) | 0.311 | <0.001 | 0.011 |
| Wood ash (W) | 0.439 | <0.001 | 0.002 |
| S×W | 0.229 | 0.489 | 0.065 |

^b^ Operation taxonomic units (97% similarity).


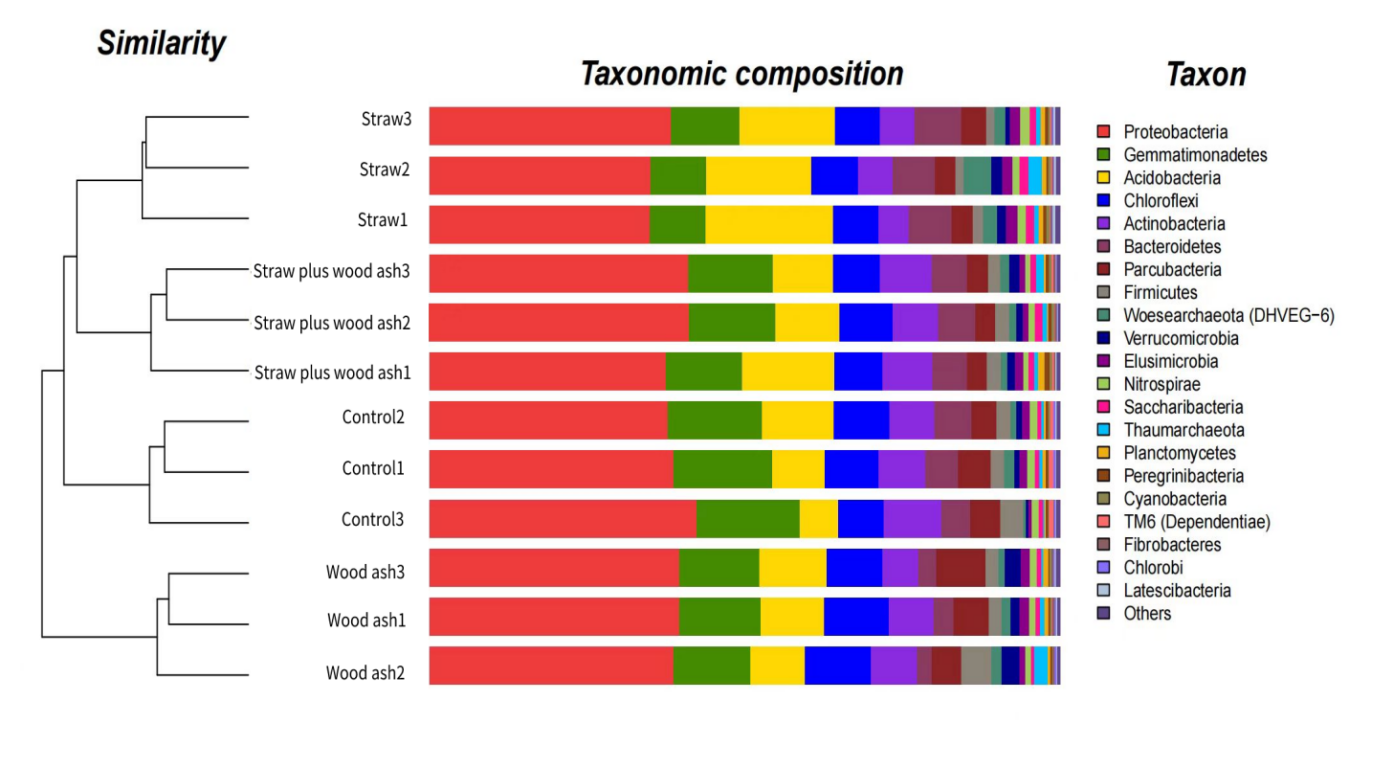


**FIGURE S1** Phylogenetic relationships of bacterial communities are shown together with the relative abundance of the dominant bacterial phyla.

**
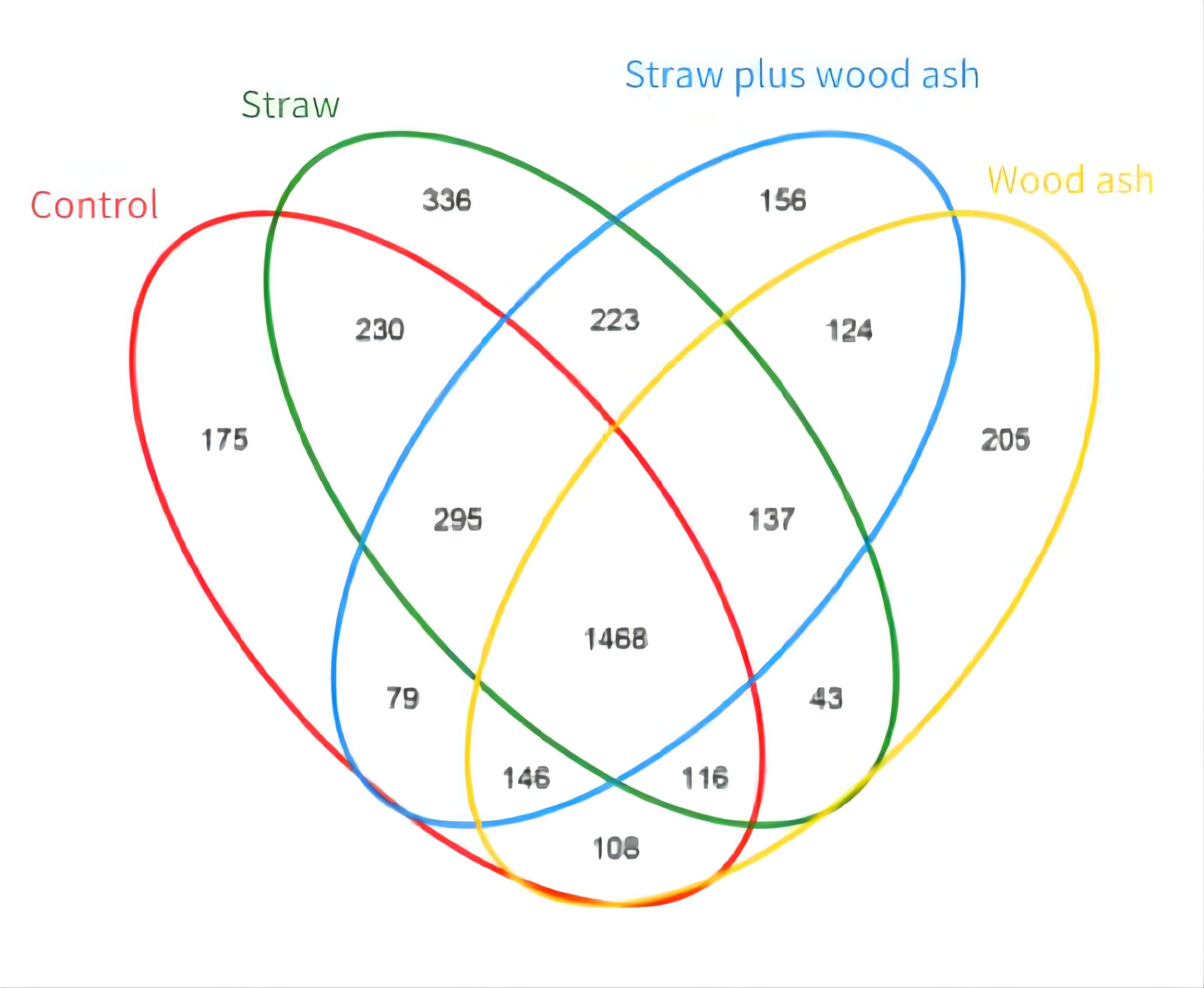
**

**FIGURE S2** A Venn diagram displaying the degree of overlap of bacterial OTUs (at the 3% evolutionary distance) among the four treatments.
